# Risk of Blood Clots After COVID-19 Vaccination and Infection: A Risk-Benefit Analysis

**DOI:** 10.21203/rs.3.rs-4378029/v1

**Published:** 2024-06-07

**Authors:** Lili Zhao, Huong Tran, Malcolm Risk, Girish Nair

**Affiliations:** University of Michigan; Corewell Health; University of Michigan; Corewell Health

**Keywords:** COVID-19 vaccine safety, Blood clots after vaccination, Blood clots after COVID-19 infection, Risk-benefit analysis

## Abstract

We analyzed the risk-benefit of COVID-19 vaccine using a causal model to explain and weigh up possible risk factors of blood clots after vaccination. A self-controlled case series method was used to examine the association between blood clots and COVID-19 vaccination. To avoid bias due to the under-reported infection among non-hospitalized subjects, a case-control study was used to compare the risk of blood clots in infected subjects to control subjects who were hospitalized due to physical injury. We found increased risks of blood clots after vaccination (incidence rate ratio is 1.13, 95% CI: [1.03,1.24] after the first dose and 1.23, 95% CI: [1.13,1.34] after the second dose). Furthermore, vaccination attenuated the increased risk of blood clots associated with infection (odds ratio is 2.16, 95% CI: [1.93,2.42] in unvaccinated versus 1.46, 95% CI: [1.25,1.70] in vaccinated). After accounting for vaccine efficacy against infection and the protection against infection-associated blood clots, receiving the COVID-19 vaccines decreases the risk of blood clots, especially during high infection rate period.

## INTRODUCTION

The Coronavirus Disease 2019 (COVID-19) pandemic prompted a race to develop and distribute effective vaccines. Approximately 81.4% of the US population has been vaccinated with at least one dose and 69.5% have completed a primary series of COVID-19 vaccination.^1^ While the benefits of vaccination are widely acknowledged, concerns have emerged regarding to the development of blood clots after vaccination.^2^ More specifically, cases of venous thromboembolism following a mRNA-based (mRNA-1273 or BNT162b2) vaccination were reported in 2022,^3–6^ drawing attention to the potential risk of blood clots after the first dose of vaccination. One study confirmed an increased risk of thromboembolism, ischemic stroke and cerebral venous sinus thrombosis after the first dose of BNT162b2,^7^ and another retrospective cohort study found an increased risk of cerebral venous thrombosis and portal vein thrombosis after any mRNA-based vaccination.^8^ Moreover, a recent systematic review^9^ has shown that thromboembolism is the most frequent adverse event following a mRNA-based vaccination. Despite those findings, vaccination is still recommended to reduce the likelihood of COVID-19 infection, hospitalization, and mortality.^7, 10^ Furthermore, COVID-19 itself substantially increases the risk of blood clots,^11–13^ implying a higher risk of mortality.^14^ Therefore, studying the risk of thromboembolic events after COVID-19 vaccination should incorporate the protective effect of vaccines against infection-associated blood clots.

The self-controlled case series (SCCS) studies conducted using various data sources have reported positive correlation between blood clots and mRNA-based vaccines, with reported incidence rate ratios (IRRs) between 1.04–1.22.^7, 15–18^ The same design was also used to evaluate the risk of blood clots after COVID-19 infection, with reported IRRs between 6.18–63.52.^7, 10, 13^ However, since a blood clot event typically requires hospitalization, subjects with blood clots are subject to a higher rate of COVID-19 testing and so at a lower likelihood of misclassification as uninfected compared to subjects without an event. Hence the SCCS design is subject to some risk of bias,^19^ which we would expect to inflate the estimated relative risk of blood clots after COVID-19 infection.

The objective of this study is to evaluate whether the overall effect of COVID-19 vaccination is to increase or decrease the risk of thromboembolic events (blood clots). To do so, we first quantified the risk of blood clots after a mRNA-based vaccine using the SCCS method. Secondly, we evaluated the association between blood clots and COVID-19infection using a case-control study, avoiding the misclassification bias associated with the SCCS method. Finally, we conducted a risk-benefit analysis by comparing the magnitude of the increased risk through the direct effect of COVID-19 vaccination with the reduced risk through the indirect pathway via protection against infection-associated blood clots.

## MATERIALS AND METHOD

### Data

Our studies used electronic health record (EHR) data from the Corewell Health East (CHE, formerly known as Beaumont Health) and Corewell Health West (CHW, formerly known as Spectrum Health) healthcare systems, which includes demographics, mortality, hospital admissions, and COVID-19infection. We obtained accurate COVID-19 vaccination records (vaccine type, date, and doses) by linking EHR data at Corewell Health with the Michigan Care Improvement Registry (MCIR), resulting in a more complete identification for individuals who received the COVID-19 vaccine outside the healthcare system. We included all patients aged ≥ 18 years old and were registered with a primary care physician within 18 months before Jan 1st, 2021.

We identified thromboembolic events by a hospital admission associated with ICD-10 (International Classification of Diseases version 10) diagnosis codes for venous thromboembolism, arterial thrombosis, cerebral venous sinus thrombosis, ischemic stroke, and myocardial infarction (Table S1). We also included patients with physical injury requiring hospitalization (list of ICD-10 codes in Table S2) as a negative control outcome to detect possible biases occurring in our studies, and further leveraged them as a control group to derive a more accurate analysis for the COVID-19 infection exposure.

### Statistical Method

We used the SCCS design to examine the association of blood clots and the first two doses of mRNA-based COVID-19 vaccines (mRNA-1273 or BNT162b2) from December 1st, 2020, to August 31st, 2022. The SCCS method compares the incidence rate of blood clots before and after vaccination. In this method, subjects are under their own control, and comparisons are made within subjects, thus avoiding any time-invariant confounding. We included subjects who had a blood clot and at least one dose of the primary series of mRNA-based vaccine in the study period. The control period was defined from December 1st, 2020, to 28 days before the first dose of vaccination, excluding the period of 28 days prior to vaccination to avoid bias due to contra-indications.^20^ Two separate risk periods for the first and second doses were defined until 28 days after vaccination, death, or August 31st, 2022, whichever occurred first (Figure S1). We also excluded subjects who had COVID-19 infection within 90 days before a thromboembolic event to remove the confounding effect of infection on that event. To ensure the independence between recurrent events, we considered only events that occurred at least one year after the previous event. We removed individuals who had incomplete covariate data and used conditional Poisson regression including an offset for the length of the risk period to obtain the IRR of blood clots after and before a COVID-19 vaccination.

In an initial analysis of the association between blood clots and COVID-19 infection, we used the same SCCS design and included patients who had at least one positive PCR or antigen test and a thromboembolic event during the period from Dec 1st, 2020, to August 31st, 2022. However, due to the missing infection data in patients who were not hospitalized for thromboembolic events or other reasons, the SCCS design results in a biased estimate of the association between blood clots and COVID-19 infection. Patients were admitted to the hospital, almost always received a COVID-19 (PCR or antigen) test, especially early in the pandemic, while patients who did not visit the hospital were subject to under-reporting of infections. This under-reporting (or misclassification of infected as uninfected) leads to an inflated IRR of blood clots after COVID-19 infection.

We proposed a simple and efficient method to quantify the association between blood clots and COVID-19 infection while dealing with the misclassification issue. The main idea is to select a subject of control (i.e., subjects without thromboembolic events) who were hospitalized for reasons independent of COVID-19 infection and therefore have complete infection data. To this end, we used patients hospitalized for physical injury as the control group, since we would not expect any causal association between physical injury and COVID-19 infection. We used a case-control design, in which an event of a blood clot is classified into the cases group, and a hospitalized injury event is considered as control group. If an individual had multiple admissions for blood clots and injury, we considered only the first admission. We determined COVID-19 infection status based on the test results during the period of 28 days prior to the date of the event (Figure S2). If an individual had a positive test result, this subject was classified as COVID-19 infected (exposed). We compared the odds of infection (exposed) vs. no infection (unexposed) in the cases (blood clots) vs. controls (physical injury) using a logistic regression model adjusted for age, race, gender, Charlson comorbidity index (CCI), number of visits, and prior vaccination status (Yes/No). Patients who had any COVID-19 vaccine between the date of positive COVID-19 test and the date event were removed. The number of visits was fit with a natural spline with three degrees of freedom. The CCI was obtained using the R package comorbidity and categorized into 4 categories, ‘0’, ‘1–2’, ‘3–4’, and ‘*≥* 5’.^21, 22^

Statistical analyses were performed in R 4.3.0. We reported OR and IRR with 95% CIs and p-values.

## RESULTS

### Study Population

#### Overall Population:

During the study period from December 1st, 2020, to August 31st, 2022, there were 747,070 subjects at Corewell Health who received mRNA-based vaccines, among which 279,229 (37.38%) had the primary series of mRNA-1273 and 467,841 (62.62%) took BNT162b2. Overall, the number of fully vaccinated patients was 711,460 (95.23%), and 35,610 (4.77%) patients received only one dose. The median age was 57 (with interquartile range [IQR]: 40–69), and 59.81% of patients were female. There were 367,105 patients taking at least one COVID-19 test (antigen or PCR), among which 78,568 (21.4%) patients received positive results. The median age was 52 (with interquartile range [IQR]: 34–67), and 61.44% of patients were female.

##### SCCS Cohort to Study Vaccination Exposure

In the study cohort of vaccination exposure, there were 18,466 patients who had at least one blood clot event and had the first dose of either mRNA-1273 or BNT162b2 vaccine. Patient demographics are presented in Table 1. We identified 3,031 events in the control period, 763 events within 28 days after the first dose, and 894 events within 28 days after the second dose. Hence, there were 4,092 patients with a total of 4,688 blood clot events considered in our conditional Poisson regression.

##### Case-control Cohort to Study Infection Exposure

49,247 patients had a hospital admission related to either blood clots or physical injury during the observational period. There were 28,304 (57.47%) patients experiencing a blood clot and 2,537 (5.15%) patients having a positive COVID-19 test result within 28 days prior to the date of the hospital admission of either blood clots or injury. Demographics of patients are presented in Table 2.

### Association Studies

#### Blood clots and mRNA-vaccination.

Based on the SCCS analysis, we found an increased risk of blood clots after the first dose (IRR: 1.13, 95% CI: [1.03, 1.24], p-value = 0.007), and after the second dose (IRR: 1.23, 95% CI: [1.13, 1.34], p-value < 0.001) of the mRNA-based vaccines.

#### Blood clots and COVID-19 exposure.

**Figure 1:**
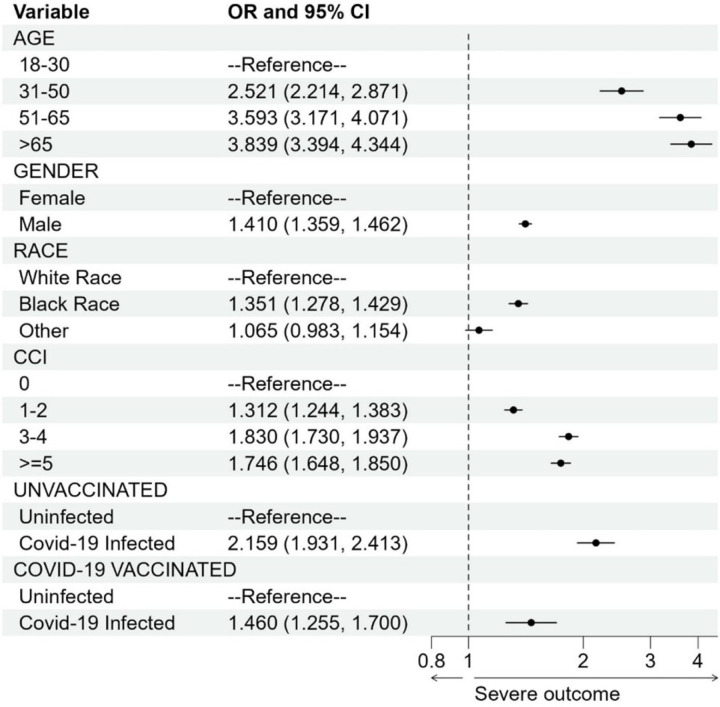
Forest plot for the case-control study to study the association between blood clots and COVID-19 infection. Odds ratio (OR) is denoted by a solid circle and 95% confidence interval (CI) is represented by a line. The x-axis is plotted on the natural log scale. CCI, Charlson Comorbidity Index.

Naïve SCCS analysis showed a very large increased risk of blood clots associated with COVID-19 infection (IRR was 19.13, 95% CI: [17.55, 20.84], p-value < 0.001). This result agrees with published IRR estimates of 6.18–63.52.^7, 10, 13^ However, a similar analysis using the hospitalized physical injury as an event also derived a large increased risk (IRR was 11.15, 95% CI: [10.13, 12.27], p-value < 0.001), indicating misclassification bias as COVID-19 infection should not substantially increase the risk of physical injury. In the case-control analysis with subjects hospitalized for physical injury as controls, we found that COVID-19 infection increased the risk of blood clots but with a much smaller magnitude than the risk in the SCCS analysis (although it is still larger than the vaccination exposure). Moreover, the degree of the increased risk was modified by vaccination status (Fig. 1). The reported odds ratio (OR) for the unvaccinated group was 2.16 (95% CI: [1.93, 2.42], p-value < 0.001) compared to 1.46 (95% CI: [1.25, 1.70], p-value < 0.001) for the vaccinated group. We observed increased risks of blood clots after COVID-19 infection in both groups, but vaccination appears to confer some protection against infection-associated thromboembolic events, given the lower OR.

#### Estimate the overall (net) effect of COVID-19 vaccination on the thromboembolic events while considering COVID-19 infection.

**Figure 2:**
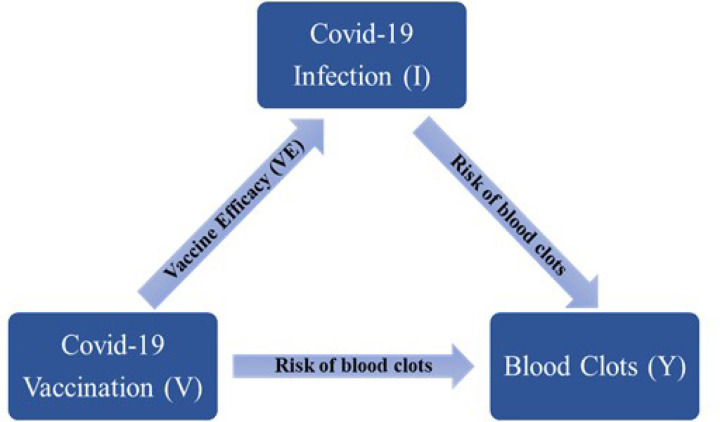
Direct and indirect associations between blood clots and mRNA-vaccines.

We extended our investigation to evaluate the overall influence of COVID-19 vaccination on the occurrence of blood clots. COVID-19 vaccines are protective against COVID-19 infection and COVID-19 severity,^23–25^ and so can indirectly decrease the likelihood of experiencing a blood clot event. Figure 2 illustrates the direct and indirect effect of the COVID-19 vaccination on the occurrence of blood clots while considering vaccine efficacy (VE). As presented in the diagram, the association between blood clots and COVID-19 vaccination is described by two paths, the direct association between blood clots and vaccination, and the indirect association between blood clots and vaccination via potential reduction in the risk of blood clots through decreasing the risk of COVID-19 infection. We estimated the overall influence of vaccination on the occurrence of blood clots by considering both direct and indirect paths.

For a vaccinated subject, the total risk is $\text{P}\left(\text{Y|V,}\;\bar{\text{I}}\right)+\text{P}\left(\text{I|V}\right)\times \text{P}\left(\text{Y|I,}\;\text{V}\right)$, where $\text{P}\left(\text{Y|V,}\;\bar{\text{I}}\right)$ is the direct risk of blood clots after vaccination given that infection has not yet occurred, and $\text{P}\left(\text{I|V}\right)\times \text{P}\left(\text{Y|I,}\;\text{V}\right)$ is the indirect risk calculated by multiplying the risk of COVID-19 infection of a vaccinated subject, $\text{P}\left(\text{I|V}\right)$, and the risk of blood clots given a COVID-19 infection in the vaccinated group, $\text{P}\left(\text{Y|I,}\;\text{V}\right)$. Similarly, the overall risk of blood clots for an unvaccinated subject is given by $\text{P}\left(\text{Y|}\bar{\text{V}}\text{,}\;\bar{\text{I}}\right)+\text{P}\left(\text{I|}\bar{\text{V}}\right)\times \text{P}\left(\text{Y|I,}\;\bar{\text{V}}\right)$. Hence the net relative risk (${\text{RR}}_{\text{Net}}$) of blood clots for a vaccinated subject compared to an unvaccinated subject is

$${\text{RR}}_{\text{Net}}=\frac{\text{P}\left(\text{Y|V,}\;\bar{\text{I}}\right)+\text{P}\left(\text{I|V}\right)\times \text{P}\left(\text{Y|I,}\;\text{V}\right)}{\text{P}\left(\text{Y|}\bar{\text{V}}\text{,}\;\bar{\text{I}}\right)+\text{P}\left(\text{I|}\bar{\text{V}}\right)\times \text{P}\left(\text{Y|I,}\;\bar{\text{V}}\right)}\;\;=\frac{\frac{\text{P}\left(\text{Y|V,}\;\bar{\text{I}}\right)}{\text{P}\left(\text{Y|}\bar{\text{V}}\text{,}\;\bar{\text{I}}\right)}+\text{P}\left(\text{I|V}\right)\times \frac{\text{P}\left(\text{Y|I,}\;\text{V}\right)}{\text{P}\left(\text{Y|I,}\;\bar{\text{V}}\right)}}{1+\text{P}\left(\text{I|}\;\bar{\text{V}}\right)\times \frac{\text{P}\left(\text{Y|V,}\;\bar{\text{I}}\right)}{\text{P}\left(\text{Y|}\bar{\text{V}}\text{,}\;\bar{\text{I}}\right)}}\;\;\text{=}\frac{{\text{RR}}_{\text{V}}\text{+P}\left(\text{I|V}\right){\text{×RR}}_{\text{IV}}}{\text{1+P}\left(\text{I|}\bar{\text{V}}\right){\text{×RR}}_{\text{I|}\bar{\text{V}}}}$$

The terms ${\text{RR}}_{\text{V}}$ is the relative risk of blood clots associated with COVID-19 vaccination, and ${\text{RR}}_{\text{I|}\bar{\text{V}}}$ is the relative risk of blood clots after a COVID-19 infection in the unvaccinated group. The term ${\text{RR}}_{\text{IV}}$ is the relative risk of blood clots in the group of subjects who have both vaccination and infection, compared to the group of subjects who do not have any exposures. Our analysis in the previous section gave an IRR of 1.23 as the measure of the association between blood clots events and the second dose of COVID-19 vaccination, therefore, we set ${\text{RR}}_{\text{V}}=1.23$. We also obtained an odd ratio ${\text{OR}}_{\text{I|}\bar{\text{V}}}=2.16$ and ${\text{OR}}_{\text{IV}}=1.49$ from the analysis using the case-control design. Since relative risk (RR) is very close to OR when the event is rare, we therefore set ${\text{RR}}_{\text{I|}\bar{\text{V}}}=2.16$ and ${\text{RR}}_{\text{IV}}=1.49$ as the blood clots is a rare event.^26^ Hence, the above ${\text{RR}}_{\text{Net}}$ becomes

$${\text{RR}}_{\text{Net}}=\frac{1.23+1.49\times \text{P}\left(\text{I}|\text{V}\right)}{1+2.16\times \text{P}\left(\text{I}|\bar{\text{V}}\right)}$$

The net relative risk of blood clots after a COVID-19 vaccination depends on the infection rate for both vaccinated and unvaccinated subjects. We defined vaccine efficacy $\text{VE}=1-\text{P}\left(\text{I|V}\right)/\text{P}\left(\text{I|}\bar{\text{V}}\right)$, then with a given infection rate for an unvaccinated subject, $\text{P}\left(\text{I|}\bar{\text{V}}\right)$, we derived the risk of infection for a vaccinated subject as $\text{P}\left(\text{I|V}\right)=\left(1-\text{VE}\right)\text{P}\left(\text{I|}\bar{\text{V}}\right)$, and obtained the net risk, ${\text{RR}}_{\text{Net}}$, as a function of VE.

**Figure 3:**
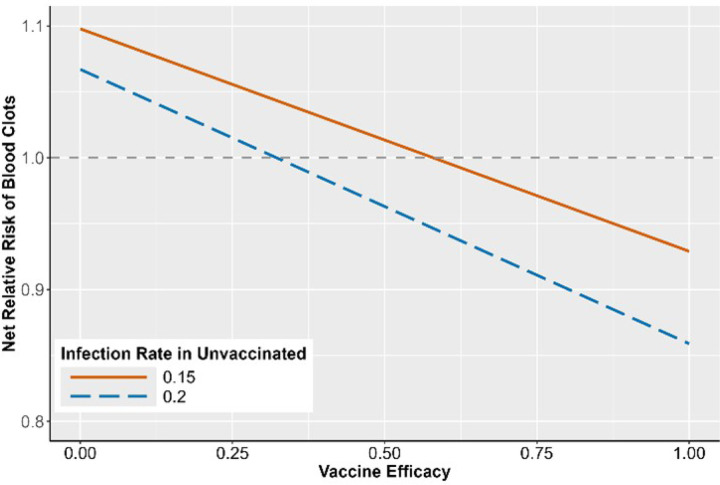
Net relative risk of blood clots comparing vaccinated and unvaccinated subjects. Infection rate 0.15 and 0.2 is the probability of COVID-19 infection before vaccination^27^. The x-axis is vaccine efficacy, and the y-axis is the net relative risk of blood clots.

Figure 3 illustrates the ${\text{RR}}_{\text{Net}}$ of blood clots after a COVID-19 vaccination as a function of VE. If ${\text{RR}}_{\text{Net}}$ is larger than one, COVID-19 vaccination increases the risk of blood blots; if ${\text{RR}}_{\text{Net}}$ is smaller than one, COVID-19 offers protection against blood clots. As VE increases from 0 to 1, ${\text{RR}}_{\text{Net}}$ decreases and reaches a point where vaccine benefits outweigh harms. In addition to VE, the infection rate in the unvaccinated population, $\text{P}\left(\text{I|}\bar{\text{V}}\right)$, also affects the ${\text{RR}}_{\text{Net}}$. During the periods with a higher infection rate, the benefit of vaccination is stronger. More specifically, given $\text{P}\left(\text{I|}\bar{\text{V}}\right)=0.2$ (a conservative estimate for early pandemic), vaccination offers protection against blood clots if VE is larger than 35%. With a VE of 80% (an estimate based on published work^27–29^), the net risk of blood clots after vaccination is reduced by 9.94% and 3.72% for $\text{P}\left(\text{I|}\bar{\text{V}}\right)=0.2$ and $\text{P}\left(\text{I|}\bar{\text{V}}\right)=0.15$, respectively.

## DISCUSSION

We found that both COVID-19 vaccination and COVID-19 infection increase the risk of blood clots. However, evidence implies that the likelihood of experiencing a thromboembolic event after COVID-19 infection is much higher than after vaccination. Our analysis agrees with previous research, indicating that COVID-19 infection is a more dangerous risk factor for blood clots than vaccination.^7, 11–13^ Different from existing work, we evaluated the association between blood clots and COVID-19 infection using a case-control study, avoiding the misclassification issue associated with the SCCS design. We also studied the effect of prior vaccination on reducing infection-associated blood clots. Moreover, we included both COVID-19 vaccination and COVID-19 infection in the analysis of the risk of blood clots and conducted a risk-benefit analysis by comparing the magnitude of the increased risk through the direct effect of COVID-19 vaccination with the reduced risk through the indirect pathway via protection against severe disease. Our analysis provides evidence that COVID-19 vaccination directly increases the risk of blood clots, but indirectly reduces the risk of infection-associated events. Results show that the indirect benefit of preventing infection-associated blood clots outweighs the direct harm if the vaccine efficacy and infection rate reach a certain level. Moreover, COVID-19 vaccination may have additional benefits in preventing blood clots associated with COVID-19 infection, as a higher rate of vaccination increases the overall level of immunity in the population, reducing the spread of the virus and conferring collective protection against infection-associated blood clots and other health risks associated with COVID-19.

Our risk-benefit analysis was conducted on the population level. This analysis can also be stratified by patient groups of interest. For example, the risk-benefit of vaccination might be different between older and younger populations.

There are several limitations to this study, mostly related to the use of EHR data from a single state. Corewell Health has 22 hospitals and the catchment area for these hospitals across many counties, hence patients may seek care at other facilities outside the Corewell Health system, leading to missing data such as infection data. To deal with the missing infection data, we used the case-control study. Moreover, the use of a prior number of hospital visits as covariates in the regression model mitigates the bias due to differing degrees of interaction with the Corewell Health system between infected and control subjects. However, our case-control design may be subjected to selection bias as subjects hospitalized with physical injury are only a subset of the control cohort and may be unrepresentative of the wider population.

Despite these limitations, our study fills an important gap in quantifying the net risk of blood clots associated with COVID-19 vaccination by considering both the direct effect of vaccination and the indirect effect through protection against COVID-19 infection and severe disease. Moreover, our work emphasizes the importance of having both indirect benefits and direct harms in consideration when studying adverse events associated with any class of vaccine. The mechanism of vaccination is to simulate the immune response the body has against infection using a dead/attenuated virus or mRNA. So, we would expect a vaccine to be associated with some side effects held in common with the virus but in a less severe form (e.g., blood clots, myocarditis,^30^ acute kidney injury^31, 32^). Therefore, studying both indirect benefits and direct harms provides a comprehensive analysis of vaccination safety.

## Supplementary Material

Supplement 1

## Figures and Tables

**Table 1:** SCCS cohort to study COVID-19 vaccination exposure on blood clots. Characteristics of patients who experienced at least one blood clot event and had at least one dose of mRNA-based vaccination from Dec 1st, 2020, to August 31st, 2022.

	Patient Characteristics in SCCS Study		
	Dose 1 (N = 18,466)	Dose 2 (N = 17,523)
**AGE**		
18–30	161 (0.87%)	142 (0.81%)
31–50	1,416 (7.67%)	1,304 (7.44%)
51–65	4,422 (23.95%)	4,166 (23.77%)
> 65	12,467 (67.51%)	11,911 (68.97%)
**GENDER**		
Female	8,691 (47.06%)	8,247 (47.06%)
Male	9,775 (52.94%)	9,276 (52.94%)
**RACE**		
White Race	14,803 (80.16%)	14,089 (80.40%)
Black Race	2,697 (14.61%)	2,514 (14.35%)
Other	966 (5.23%)	920 (5.25%)
**CHARLSON COMORBIDITY INDEX (CCI)**		
0	2,938 (15.91%)	2,801 (15.98%)
1–2	5,153 (27.91%)	4,910 (28.02%)
3–4	5,326 (28.84%)	5,061 (28.88%)
>=5	5,049 (27.34%)	4,751 (27.11%)
**NUM. BLOOD CLOTS IN RISK PERIODS**		
	763	894

**Table 2:** Case-control cohort to study COVID-19 infection exposure on blood clots Characteristics of patients who experienced a blood clot or hospitalized injury event from Dec 1st, 2020, to August 31st, 2022.

Patient Characteristics in Case-control Study			
	Blood clots N = 28,304 (57.47%)	Physical injury N = 20,943 (42.53%)	Overall N = 49,247
**AGE**			
18–30	378 (1.34%)	1,118 (5.33%)	1,496 (3.04%)
31–50	2,686 (9.49%)	2,902 (13.86%)	5,588 (11.35%)
51–65	7,280 (25.72%)	5,184 (24.75%)	12,464 (25.31%)
> 65	17,960 (63.45%)	11,739 (56.05%)	29,699 (60.31%)
**GENDER**			
Female	13,207 (46.66%)	11,588 (55.34%)	24,795 (50.35%)
Male	15,097 (53.34%)	9,355 (44.67%)	24,452 (49.65%)
**RACE**			
White Race	22,568 (79.73%)	17,135 (81.8%)	39,703 (80.62%)
Black Race	4,175 (14.75%)	2,577 (12.30%)	6,752 (13.71%)
Other	1,561 (5.52%)	1,231 (5.88%)	2,792 (5.67%)
**CHARLSON COMORBIDITY INDEX (CCI)**			
0	4,722 (16.68%)	5,389 (25.73%)	10,111 (20.53%)
1–2	7,957 (28.11%)	6,469 (30.89%)	14,426 (29.29%)
3–4	8,047 (28.43%)	4,535 (21.65%)	12,582 (25.55%)
>=5	7,578 (26.77%)	4,550 (21.73%)	12,128 (24.63%)
**COVID-19 VACCINATION BEFORE INFECTION**			
NO	12,313 (43.50%)	9,341 (44.60%)	21,654 (43.97%)
YES	15,991 (56.50%)	11,602 (55.40%)	27,593 (56.03%)
**INFECTION WITHIN 28 DAYS PRIOR TO AT THE DATE OF EVENT**			
NO	26,510 (93.66%)	20,200 (96.45%)	46,710 (94.85%)
YES	1,794 (6.34%)	743 (3.55%)	2,537 (5.15%)

## Data Availability

The datasets analyzed during the current study are not publicly available due to privacy or ethical restrictions but are available from the corresponding author on reasonable request.

## References

[1] Centers for Disease Control and Prevention. COVID Data Tracker. Atlanta, GA: U.S. Department of Health and Human Services, CDC; https://covid.cdc.gov/covid-data-tracker. Nov 17, 2023.

[2] Mani Avinash, Ojha Vineeta. Thromboembolism after COVID-19 Vaccination: A Systematic Review of Such Events in 286 Patients. Annals of Vascular Surgery; May 2022; Volume 84(12–20.e1). (10.1016/j.avsg.2022.05.001). PMC9093198 35568325

[3] Dias Leonor, Soares-dos-Reis Ricardo, Joao Meira, Cerebral venous thrombosis after BNT162b2 mRNA SARS-CoV-2 vaccine. Journal of Stroke and Cerebrovascular Diseases; May 202; Volume 30(8). (10.1016/j.jstrokecerebrovasdis.2021.105906). PMC8148614 34111775

[4] Andraska Elizabeth A., Kulkarni Rohan, Mirnal Chaudhary M, Sachdev Ulka. Three cases of acute venous thromboembolism in females following vaccination for COVID-19. Journal of Vascular Surgery: Venous and Lymphatic Disorders; Jan 2021; Volume 10(1). (10.1016/j.jvsv.2021.07.009). PMC8327605 34352418

[5] Carli Giuseppe, Nichele Ilaria, Ruggeri Marco, Barra Salvatore, Tosetto Alberto. Deep vein thrombosis (DVT) occurring shortly after the second dose of mRNA SARS-CoV-2 vaccine. Intern Emerg Med; Mar 2021; Volume 16. (10.1007/s11739-021-02685-0). PMC7940863 33687691

[6] Zakaria Zaitun, Asma Sapiai Nur, Rahman Izaini Ghani Abdul. Cerebral venous sinus thrombosis 2 weeks after the first dose of mRNA SARS-CoV-2 vaccine. Acta Neurochir; Jun 2021; 163, 2359–2362. (10.1007/s00701-021-04860-w). 34101024 PMC8186353

[7] Hippisley-Cox Julia, Patone Martina, Mei Xu W, Risk of thrombocytopenia and thromboembolism after covid-19 vaccination and SARS-CoV-2 positive testing: self-controlled case series study. BMJ; August 2021; 374. (10.1136/bmj.n1931). PMC8388189 34446426

[8] Taquet Maxime, Husain Masud, Geddes John R, Luciano Sierra, Harrison Paul J. Cerebral venous thrombosis and portal vein thrombosis: A retrospective cohort study of 537,913 COVID-19 cases. eClinicalMedicine; Volume 39(101061); July 2021. (10.1016/j.eclinm.2021.101061). PMC8324974 34368663

[9] Yasmin Farah, Najeeb Hala, Naeem Unaiza, Adverse events following COVID-19 mRNA vaccines: A systematic review of cardiovascular complication, thrombosis, and thrombocytopenia. Immunity, Inflammation and Disease; 2023; Volume 11(3). (10.1002/iid3.807). PMC10022421 36988252

[10] Ho Frederick K., Man Kenneth K.C., Toshner Mark, Thromboembolic Risk in Hospitalized and Nonhospitalized COVID-19 Patients: A Self-Controlled Case Series Analysis of a Nationwide Cohort. Mayo Clinic Proceedings; Vol. Volume 96(10); pp. 2587–2597. (10.1016/j.mayocp.2021.07.002). PMC8282478 34607634

[11] Xiong Xiaoming, Chi Jianhua, and Gao Qinglei. Prevalence and risk factors of thrombotic events on patients with COVID-19: a systematic review and meta-analysis. Thrombosis Journal; 2021; Volume 19(32). (10.1186/s12959-021-00284-9). PMC8132033 34011381

[12] Schulman Sam, Hu Yu, and Konstantinides Stavros. Venous Thromboembolism in COVID-19. Thrombosis and Haemostasis; 2020; Volume 120(12). (10.1055/s-0040-1718532). PMC7869046 33099284

[13] Katsoularis I, Fonseca-Rodríguez O, Farrington P, Risk of acute myocardial infarction and ischaemic stroke following COVID-19 in Sweden: a self-controlled case series and matched cohort study. The Lancet; 2021; Volume 398(10300). (10.1016/s0140-6736(21)00896-5). PMC8321431 34332652

[14] Malas Mahmoud B., Naazie Isaac N., Elsayed Nadin, Thromboembolism risk of COVID-19 is high and associated with a higher risk of mortality: A systematic review and meta-analysis. eClinicalMedicine; 2020; Volume 29(100639). (10.1016/j.eclinm.2020.100639). PMC7679115 33251499

[15] Simpson C.R., Shi T., Vasileiou E., First-dose ChAdOx1 and BNT162b2 COVID-19 vaccines and thrombocytopenic, thromboembolic and hemorrhagic events in Scotland. Natural Medicine, Volume 29(7); 1290–1297; 2021. (10.1038/s41591-021-01408-4). PMC8282499 34108714

[16] Joelle Jabagi Marie, Botton Jérémie, Bertrand Marion, Myocardial Infarction, Stroke, and Pulmonary Embolism After BNT162b2 mRNA COVID-19 Vaccine in People Aged 75 Years or Older. JAMA; 2022; Volume 327(1): 80–82. (10.1001/jama.2021.21699). 34807248 PMC8609457

[17] Dag Berild Jacob, Bergstad Larsen Vilde, Myrup Thiesson Emilia, Analysis of Thromboembolic and Thrombocytopenic Events After the AZD1222, BNT162b2, and MRNA-1273 COVID-19 Vaccines in 3 Nordic Countries. JAMA Netw Open; 2022; Volume 5(6). (10.1001/jamanetworkopen.2022.17375). PMC9198750 35699955

[18] Sze Ling Chui Celine, Fan Min, Fai Wan Eric Yuk, Thromboembolic events and hemorrhagic stroke after mRNA (BNT162b2) and inactivated (CoronaVac) covid-19 vaccination: A self-controlled case series study. eClinicalMedicine, 2022; Vol. 50(101504). (10.1016/j.eclinm.2022.101504). PMC9233170 35770253

[19] Griffith Gareth J., Morris Tim T., Tudball Matthew J., Collider bias undermines our understanding of COVID-19 disease risk and severity. Nature communications; 2020; Volume 11(1). (10.1038/s41467-020-19478-2). PMC7665028 33184277

[20] Fan Bu, Schuemie Martijn J., Nishimura Akihiko, Bayesian safety surveillance with adaptive bias correction. Statistics in Medicine; 2023; Volumn 43(2); 395–418. (10.1002/sim.9968). 38010062

[21] Charlson ME, Pompei P, Ales KL, MacKenzie CR. A new method of classifying prognostic comorbidity in longitudinal studies: development and validation. Journal of Chronic Diseases; Volume 40(5). (10.1016/0021-9681(87)90171-8). 3558716

[22] Gasparini Alessandro. An R package for computing comorbidity scores. Journal of Open Source Software; 2018; Volumn 3(23); p. 648.

[23] Fleming-Dutra KE, Britton A, Shang N, Association of Prior BNT162b2 COVID-19 Vaccination With Symptomatic SARS-CoV-2 Infection in Children and Adolescents During Omicron Predominance. JAMA; 2022; Volume 327(22). (10.1001/jama.2022.7493). PMC9107063 35560036

[24] Fleming-Dutra Katherine E., Avrich Ciesla Allison, Roper Lauren E., Preliminary Estimates of Effectiveness of Monovalent mRNA Vaccines in Preventing Symptomatic SARS-CoV-2 Infection Among Children Aged 3–5 Years — Increasing Community Access to Testing Program, United States, July 2022-February 2023. Morbidity and Mortality Weekly Report; 2023;72:177–182. (10.15585/mmwr.mm7207a3). 36795625 PMC9949847

[25] Hatfield Kelly M, Baggs James, Wolford Hannah, Effectiveness of Coronavirus Disease 2019 (COVID-19) Vaccination Against Severe Acute Respiratory Syndrome Coronavirus 2 (SARS-CoV-2) Infection Among Residents of US Nursing Homes Before and During the Delta Variant Predominance. Clinical Infectious Diseases; 2022; Volume 75(Supplementary 2). (10.1093/cid/ciac562). PMC9384512 35856635

[26] Silverstein MD, Heit JA, Mohr DN, Petterson TM, O’Fallon WM, Melton LJ 3rd. Trends in the incidence of deep vein thrombosis and pulmonary embolism: a 25-year population-based study. Arch Intern Med; 1999; Volume 158(6). (10.1001/archinte.158.6.585). 9521222

[27] Shen Chen, Risk Malcolm, Schiopu Elena, Efficacy of COVID-19 vaccines in patients taking immunosuppressants. Ann Rheum Dis; 2022; Volume 81(6): 875–880. (10.1136/annrheumdis-2021-222045). 35197265 PMC9422955

[28] Risk Malcolm, Shen Chen, Salim S Hayek, et tal. Comparative Effectiveness of Coronavirus Disease 2019 (COVID-19) Vaccines Against the Delta Variant. Clin Infect Dis. 2022; Volume 75(1):e623–e629. (10.1093/cid/ciac106). 35137006 PMC9047165

[29] Risk Malcolm, Hayek Salim S, Schiopu Elena, COVID-19 vaccine effectiveness against omicron (B.1.1.529) variant infection and hospitalisation in patients taking immunosuppressive medications: a retrospective cohort study. The Lancet Rheumatology; Volume 4(11), e775–e784. (10.1016/S2665-9913(22)00216-8). PMC9381025 35991760

[30] Patone Martian, Xu W Mei Lahiru Handunnetthi, Risk of Myocarditis After Sequential Doses of COVID-19 Vaccine and SARS-CoV-2 Infection by Age and Sex. Circulation; 2022; Volume 146(10); (10.1161/circulationaha.122.059970). PMC9439633 35993236

[31] Luo Huiting, Li Xiaolin, Ren Qidong, at el. Acute kidney injury after COVID-19 vaccines: a real-world study. Renal Failure; 2022; Volume 44(1). (10.1080/0886022x.2022.2081180). PMC9196826 35678258

[32] Fabrizio Fabrizi, Alfieri Carlo M., Cerutti Roberta, COVID-19 and Acute Kidney Injury: A Systematic Review and Meta-Analysis. Pathogens. 2020; 9(12):1052. (10.3390/pathogens9121052). 33334023 PMC7765425

